# POEMS syndrome characterized by bone lesions: A case report

**DOI:** 10.1097/MD.0000000000036678

**Published:** 2023-12-15

**Authors:** Yang Wang, Yalong Liu, Xiaoli Huang, Lan Peng, Zhijun Zhang

**Affiliations:** a Wenjiang District People’s Hospital, Chengdu, China; b West China Hospital of Sichuan University, Chengdu, China.

**Keywords:** bone lesions, M protein profile, POEMS syndrome

## Abstract

**Rationale:**

POEMS (polyneuropathy, organomegaly, endocrinopathy, monoclonal paraproteinemia, and skin changes) syndrome is a rare clinical syndrome characterized by multiple peripheral neuropathies, hepatosplenomegaly, endocrine disorders, monoclonal paraproteinemia, and dermatosis. The main manifestations of POMES were nerve and skin changes, and bone disease was not reported. Here, we report a case of POEMS syndrome with the main manifestation of bone lesions.

**Patient concerns:**

POMES is rare and its clinical manifestations are complex, making it difficult for patients to find the department they should visit. It is easy to miss diagnosis and misdiagnosis, delay the treatment time of patients, and affect the prognosis.

**Diagnosis interventions:**

The patient was admitted to the gastroenterology department due to hepatic insufficiency. Multiple osteogenic changes were found by improved enhanced CT due to screening for causes of hepatic insufficiency, and spleen enlargement was indicated by abdominal ultrasound. Due to the involvement of multiple system problems, and follow-up of medical history, it was found that there was a history of discoloration of the distal limb in cold weather in the past 5 years. All things considered, it may be POMES. Further refinement of the bone marrow examination revealed active proliferation of granulocytes and erythrocytes. Bone marrow biopsy showed active hyperplasia, dominated by granulocytes. IFE showed IgA (type λ) and monoclonal myeloma (M) protein bands. To sum up, POMES diagnosis is considered.

**Outcomes:**

After the diagnosis is clear and the informed consent of the patient and his family is obtained, prednisone acetate is anti-inflammatory, lenalidomide is used to regulate immune function, liver and stomach protection treatment and bile secretion promotion are given. The patient reported improvement in liver function, significant improvement in overall and limb stiffness, and was discharged with improvement.

**Lessons:**

Although bone lesions are not typically the main manifestation of POEMS syndrome, this diagnosis should be considered when this manifestation is combined with organ enlargement, skin changes, and peripheral neuropathy. In addition, the collection of medical history is crucial, when there is a clinical manifestation and auxiliary examination does not match, the idea should be expanded according to the relevant evidence, and finally make the corresponding diagnosis.

## 1. Introduction

POEMS syndrome, also known as Crow–Fukase syndrome, is a rare multi-system disorder associated with abnormal immunoglobulin production by plasma cells. It typically begins in middle age and affects more males than females.^[1]^ The natural course of POEMS syndrome consists mainly of the progression of peripheral neuropathy (present in nearly 100% of patients)^[2]^ to cause bedriddenness, and common causes of death are heart failure and lung infection. The early diagnosis of the disease is difficult, and the risk of death is high. However, the 5-year survival rate can reach 85% with aggressive treatment. The etiology of POEMS syndrome remains unclear; recent work suggests that its pathogenesis is related to vascular endothelial growth factor (VEGF), proinflammatory cytokines, matrix metalloproteinases, and human herpes virus 8 infection.^[3,4]^ The acronym “POEMS” summarizes the main clinical manifestations of this syndrome: polyneuropathy (including numbness and tingling in the distal extremities), organomegaly (including hepatosplenomegaly), endocrinopathy, myeloma/monoclonal proteinemia, and skin changes. Other characteristic features of POEMS syndrome include high levels of serum endothelial growth factor, monoclonal plasma cell hyperplasia, papilledema, bone lesions, and hemangioma.^[5]^ Due to the complexity and diversity of the syndrome, however, patients are often referred to different departments according to the initial or main manifestations with which they present. The multi-system involvement and rarity of POEMS syndrome lead to a strong possibility of missed diagnosis and misdiagnosis. The syndrome may be misdiagnosed as a neuropathy, such as Guillain–Barre syndrome, or as tuberculosis, diabetes, chronic nephritis, various skin diseases, and multiple myeloma (MM).^[6]^ Early diagnosis and treatment can significantly improve the prognosis of this syndrome. Here, we report a case of POEMS syndrome with bone lesions as the main manifestation, which to our knowledge has not been reported previously.

The diagnostic criteria for POEMS syndrome are shown in Table 1.

**Table 1:** Diagnostic criteria for POEMS syndrome.

Diagnostic criteria	Clinical manifestation
Mandatory main standards	1. Multiple peripheral neuropathy
	2. Monoclonal plasma cell dysplasia
Main standards	1. Castleman disease
	2. Sclerosing osteopathy
	3. Elevated blood VEGF level
Secondary criteria	1. Organ enlargement (splenomegaly, hepatomegaly or lymph node enlargement)
	2. Edema (limb edema, pleural effusion or ascites)
	3. Endocrine diseases (adrenal gland, thyroid gland or pancreas, etc.)
	4. Skin changes (pigmentation, hirsutism)
	5. Papilledema
	6. Thrombocytosis
	7. Polycythemia

Diagnostic requirements: 2 mandatory main standards + 1 main standard + 1 secondary criterion.

## 2. Case presentation

A 52-year-old woman was admitted to our hospital for the treatment of hepatic insufficiency discovered 1 day previously. She reported taking a cold medicine due to flu-like symptoms, after which she experienced fatigue, anorexia to oily food, and yellow urine. A liver function test showed that the patient’s alanine aminotransferase (ALT) level was 869 U/L and her aspartate aminotransferase (AST) level was 739 U/L. The patient reported that she had experienced 2 episodes of left lower abdominal discomfort, which improved after 1 hour of hot compress use. A review of other systems was unremarkable.

The patient had a history of migraine, for which she had taken oral gastrodin over the past 2 years. An abdominal ultrasound performed 1 year previously revealed splenomegaly (length, 4.9 cm). The patient’s body temperature was 36.6°C, pulse rate was 88 beats/min, respiration rate was 20 breaths/min, and blood pressure was 136/80 mm Hg. Breath sounds in both lungs were clear, with no dry or wet rale. The patient’s whole abdomen was soft, with no tenderness, rebound tenderness, abdominal muscle tension, or abnormal bowel sound.

A biochemical workup yielded the following results: ALT level, 935 U/L; AST level, 596 U/L; alkaline phosphatase (ALP) level, 513 U/L; gamma-glutamyl transferase level, 348 U/L; thiobarbituric level, 15.7 μmol/L; and triglyceride level, 2.41 mmol/L. The patient’s VEGF level was 710.12 pg/mL, and immunological tests revealed an immunoglobulin (Ig) A level of 5670 mmol/L and normal IgG and IgM levels. The rheumatoid factor level was 29.40 IU/mL, and the complement C3 and C4 levels were 0.629 and 0.144 g/L, respectively. Other tests, including a complete blood count; the evaluation of coagulation function, the D-dimer, glycosylated hemoglobin, myocardial markers, blood amylase, and cold cell and cold agglutination; B-type natriuretic peptide, tuberculosis antibody, and purified protein derivative tests; and a complete immune set with the assessment of antineutrophil cytoplasmic antibodies, testosterone, progesterone, estradiol, luteinizing hormone, follicle-stimulating hormone, and prolactin, yielded unremarkable results.

**Figure 1.**
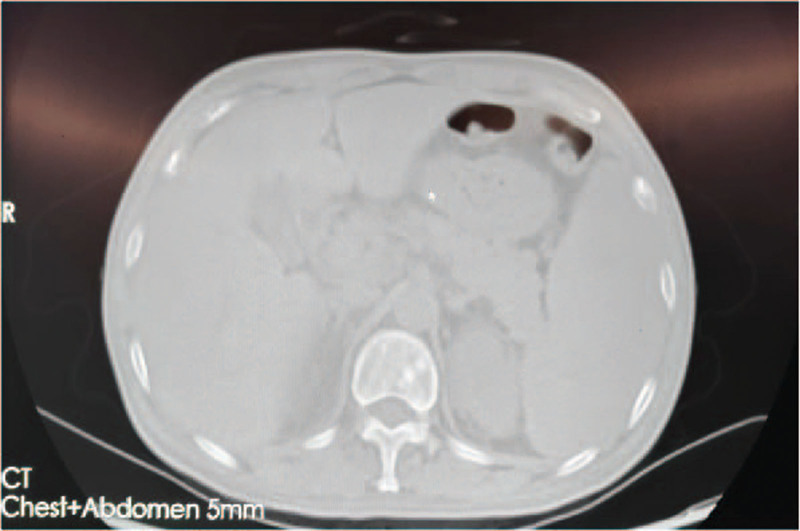
Multiple bilateral osteogenic changes in the enhanced CT.

Enhanced CT revealed multiple bilateral osteogenic changes (Fig. 1) in the ribs, sternum, clavicles, and scapulae; multiple vertebral bodies and the pelvis were also affected. Lymphadenopathy was present in both axillae. The numbers of lymph nodes in the mesentery and retroperitoneum were increased, with partial enlargement indicating the possibility of tumor metastasis. Splenomegaly was also observed. A PET-CT examination in outer court performed high-density nodular shadows scattered in many bones, including the bilateral scapulae and clavicles, left humeral head, multiple bilateral ribs, multiple vertebral bodies and appendages of the whole spine, sternum, pelvis, and bilateral upper femoral segments. Active fluorodeoxyglucose (FDG) metabolism suggesting bone metastasis was observed. Multiple lymph nodes were seen under the chin and in the bilateral submaxillary and carotid sheaths, posterior cervical space, supra- and infra-clavicular fossae, and axillae. A few lymph nodes in the mediastinum and bilateral hilum and multiple lymph nodes around the abdominal aorta and in the upper segment of the mesentery, bilateral external iliac arteries, and groin showed slightly increased FDG metabolism. No active FDG metabolism was associated with the splenomegaly.

Due to the discovery of bone lesions, spleen enlargement, and other problems involving multiple systems, we conducted a bone marrow biopsy and immunoglobulin and other examinations. Bone marrow examination revealed the active proliferation of granulocyte and erythrocyte lineages. A bone marrow biopsy showed active hyperplasia with predominance of the granulocyte lineage. Immunofixation electrophoresis (IFE) revealed IgA (λ type) and monoclonal myeloma (M) protein bands.

Liver-protective treatment was administered during the patient’s hospitalization. During the workup period, enhanced CT revealed multiple osteogenic changes, lymphadenopathies, and serous cavity effusions. Thyroid function test results were abnormal and skin hyperpigmentation was observed. In view of the multi-system involvement of the patient’s clinical manifestations and the test results, we considered that the bone lesions might be attributable POEMS syndrome. This diagnosis was further supported by the patient’s reporting of a history of distal extremity discoloration during cold weather for the past 5 years, in addition to paresthesia in the legs and numbness and tingling in a glove pattern over the past 6 months. The diagnosis of POEMS syndrome was confirmed by further examinations, including (but not limited to) immunology, electromyography, VEGF measurement, IFE, PET-CT, and bone marrow biopsy, which revealed polyneuropathy, monoclonal M protein expression, sclerosing bone disease, lymphadenopathy, skin changes, multiple serous cavity effusions, and an increased blood VEGF level.

In addition to liver- and gastro-protective treatment and the promotion of bile secretion, the patient was given 20 mg oral prednisone acetate twice daily for the treatment of POEMS syndrome. The patient reported significant improvement overall and in her limb stiffness. After communicating to the patient and her relatives about the efficacy and side-effect profile of lenalidomide, and obtaining their written informed consent, a daily oral dose of 25 mg lenalidomide was added to the treatment plan. After the first course of treatment (1 week), the patient experienced no bone marrow suppression or significant discomfort. The patient’s condition improved, and she was discharged.

## 3. Discussion

The diagnosis of POEMS syndrome remains challenging due its rarity and multiple clinical presentations. In this case, the patient was admitted to hospital due to liver insufficiency, caused mainly by drug-induced liver injury, but multiple bone lesions were unexpectedly found during her hospitalization. Relevant examinations were performed and POEMS syndrome was diagnosed. Although the diagnostic criteria for POEMS syndrome include osteosclerosis, this disease is rare and manifests mostly as skin lesions and neuropathy.

The clinical diagnosis of POEMS syndrome in the present case was supported by the patient’s multiple peripheral neuropathy (as detected by electromyography of the upper and lower limbs), M protein profile (λ type-I IgA and M protein monoclonal bands were detected by IFE), sclerotic bone disease (reflected by an osteogenic ALP increase, high-density osteogenesis on chest and abdominal CT examinations, and suggestion of bone metastasis on PET-CT images), lymphadenopathy (reflected by bilateral axillary lymph-node enlargement and increased numbers and partial enlargement of mesenteric and retroperitoneal lymph nodes on CT), skin changes (distal limb discoloration when cold, skin hyperpigmentation), edema or pleural effusion and ascites (reflected by multiple serous cavity effusion on CT), and an increased VEGF level. In the present case, the main clinical manifestation of POEMS syndrome was bone lesions, defined as the replacement of the original bone structure by inflammatory, neoplastic, or other pathological tissues.^[7]^ The observation of the shape, margins, size, and number of bone lesions lesions provides insights into qualitative diagnosis. Bone lesions is related to the activity of interleukin-1, prostaglandin, tumor necrosis factor, and other cytokines, as well as osteoclasts.^[8]^ In this case, we considered the differential diagnoses of MM and bone tuberculosis based on the observed bone lesions.^[9]^ Most patients with MM present with the initial symptom of bone pain, unlike the typical presentation of POEMS syndrome. In addition, MM occurs later in life than does POEMS syndrome, and hepatosplenomegaly is common whereas lymphadenopathy is rare. An increased erythrocyte sedimentation rate, elevated blood calcium level, and the presence of M and urinary Bence–Jones proteins are significantly more common in MM than in POEMS syndrome. X-rays of MM usually show osteoporosis, multiple sites of osteolytic lesions, and rare osteosclerotic bone lesions. In addition, bone marrow biopsy shows a significant increase in the number of plasma cells. In the present case, examination suggested that the bone lesions was osteosclerotic. Multiple lymphadenopathies were present, and no plasma cells were found on bone marrow aspiration, ruling out the diagnosis of MM. In bone tuberculosis, the metabolism of the local bone tissue ceases and the blood supply to the bone is interrupted, resulting in bone necrosis. X-rays of this disease show localized increases in bone density. In contrast, the majority of patients with POEMS syndrome have bone lesions, most (54–82%) of which are osteosclerotic.^[10]^ Moreover, our patient showed no tuberculosis-related symptom such as fever, night sweats, or fatigue. Given that the patient had no history of tuberculosis and CT examination revealed disseminated high-density osteogenic changes, rather than localized increases in density, we ruled out tuberculous-induced bone lesions. Taken together, the multi-system lesions, pleural effusion, ascites, peripheral neuropathy, and M proteinemia in this patient, as well as her 5-year history of distal extremity discoloration in cold weather, supported the conclusion that the bone lesions was a manifestation of POEMS syndrome. After the clear establishment of a diagnosis and 1 week of treatment, the patient improved significantly and was discharged. POEMS syndrome is commonly missed due to its rarity and varied clinical presentations. This case suggests that the diagnosis of POMES syndrome should be considered when a patient presents with unexplained bone lesions, and that early treatment and intervention are critical to improve the prognosis.

## Acknowledgments

We thank *Medjaden* Inc. for scientific editing of this manuscript.

## Author contributions

**Conceptualization:** Yang Wang, Yalong Liu, Xiaoli Huang, Lan Peng, Zhijun Zhang.

**Data curation:** Yang Wang, Yalong Liu, Xiaoli Huang, Lan Peng, Zhijun Zhang.

**Formal analysis:** Yang Wang, Yalong Liu, Xiaoli Huang, Lan Peng, Zhijun Zhang.

**Visualization:** Yang Wang, Yalong Liu, Xiaoli Huang, Lan Peng, Zhijun Zhang.

**Writing – original draft:** Yang Wang, Yalong Liu, Xiaoli Huang, Lan Peng, Zhijun Zhang.

**Writing – review & editing:** Yang Wang, Yalong Liu, Xiaoli Huang, Lan Peng, Zhijun Zhang.

## References

[1] Kim DE Kim HJ Kim YA. Kaposi’s sarcoma herpesvirus-associated Castleman’s disease with POEMS syndrome. Muscle Nerve. 2000;23:436–9. 10679723 10.1002/(sici)1097-4598(200003)23:3<436::aid-mus18>3.0.co;2-i

[2] Li J Zhou DB Huang Z. Clinical characteristics and long-term outcome of patients with POEMS syndrome in China. Ann Hematol. 2011;90:819–26. 21221584 10.1007/s00277-010-1149-0

[3] Scarlato M Previtali SC Carpo M. Polyneuropathy in POEMS syndrome: role of angiogenic factors in the pathogenesis. Brain. 2005;128:1911–20. 15975949 10.1093/brain/awh519

[4] Rosti V Massa M Campanelli R. Vascular endothelial growth factor promoted endothelial progenitor cell mobilization into the peripheral blood of a patient with POEMS syndrome. Haematologica. 2007;92:1291–2. 17768134 10.3324/haematol.11455

[5] Watanabe O Arimura K Kitajima I. Greatly raised vascular endothelial growth factor (VEGF) in POEMS syndrome. Lancet. 1996;347:702. 10.1016/s0140-6736(96)91261-1 8596427

[6] Gertz MA Comenzo R Falk RH. Definition of organ involvement and treatment response in immunoglobulin light chain amyloidosis (AL): a consensus opinion from the 10th International Symposium on Amyloid and Amyloidosis, Tours, France, 18-22 April 2004. Am J Hematol. 2005;79:319–28. 16044444 10.1002/ajh.20381

[7] Tateiwa D Yoshikawa H Kaito T. Cartilage and bone destruction in arthritis: pathogenesis and treatment strategy: a literature review. Cells. 2019;8:818. 31382539 10.3390/cells8080818 PMC6721572

[8] Zhang B Song X Liang B. The clinical study of POEMS syndrome in China. Neuro Endocrinol Lett. 2010;31:229–37. 20424579

[9] Brigle K Rogers B. Pathobiology and diagnosis of multiple myeloma. Semin Oncol Nurs. 2017;33:225–36. 28688533 10.1016/j.soncn.2017.05.012

[10] Dispenzieri A. POEMS syndrome. Hematology Am Soc Hematol Educ Program. 2005;2005:360–7. 10.1182/asheducation-2005.1.360 16304404

